# Does laparoscopy still has a role in modern fertility practice?

**Published:** 2017-12

**Authors:** Ahmad Mahran, Ahmed R Abdelraheim, Abdelrahman Eissa, Mohamed Gadelrab

**Affiliations:** *Department of Obstetrics and Gynecology, Faculty of Medicine, Minia University, Minia, Egypt.*

**Keywords:** Infertility, Laparoscopy, Hysteroscopy

## Abstract

**Background:**

More than 3 decades after the introduction of in vitro fertilization (IVF) and despite the improved success rates of assisted reproductive technologies, the argument for performing laparoscopy as a part of the infertility workup still stands.

**Objective:**

To evaluate the role of laparoscopy±hysteroscopy in diagnosis and management of infertility in our setting in view of modern fertility practice.

**Materials and Methods:**

This case control study was carried out on 600 infertile women subjected to laparoscopy or combined laparoscopy and hysteroscopy at endoscopy unit in Minia University Hospital, Egypt during the period from January 2012 to December 2014.

**Results:**

The causes of infertility as identified by laparoscopy±hysteroscopy were polycystic ovary syndrome (25.1%), tubal factor (30%), uterine cause (4%), and endometriosis (2.7%). No cause was identified in 38.2% of cases. Based on operative findings, women were treated with different options. Expectant management was used in 92 cases (15.3%). Ovulation induction with anti-estrogens or gonadotropins was used in 372 cases (62%). Sixty cases (10%) had intrauterine insemination and sixty four cases (10.7%) underwent in vitro fertilization (IVF) / intracytoplasmic sperm injection (ICSI) treatment. Within 1 yr after laparoscopy, 180 cases achieved pregnancy (30%). The most favorable outcome was recorded in women with unexplained infertility (36.7% of cases got pregnant) followed by women with polycystic ovary syndrome (27.8%). Participants with uterine and tubal infertility factor achieved pregnancy in 25% and 22.8% of cases, respectively. The worst outcome was recorded in women with endometriosis.

**Conclusion:**

Laparoscopy still has an important role in the diagnosis and treatment of infertility.

## Introduction

Infertility is defined as failure to conceive after 24 months of regular unprotected sexual intercourse in the absence of known reproductive pathology (1). It is prevalent as one in seven couples (2-9). Infertility is not just a medical problem, but many of those failing to conceive deal with medical, psychological and financial stresses related to their condition (10). More than 3 decades after the introduction of in vitro fertilization (IVF) and despite the improved success rates of assisted reproductive technologies (ART), the argument for performing laparoscopy as a part of the infertility workup still stands (11). It is agreed that the use of laparoscopy in women with decreased ovarian reserve or severe male factor infertility offers no benefit since the main treatment will remain IVF. The major controversy remains in women with endometriosis, tubal adhesions, history of tubal sterilisation, and uterine fibroids distorting the uterine cavity (12). Unexplained infertility remains a challenge in diagnosis and management even after the introduction of IVF technology. It is well known that patency of the fallopian tubes by hysterosalpingogram (HSG) does not rule out pelvic adhesions that can only be diagnosed by laparoscopy. It is debatable whether laparoscopy should be a mandatory step in sub-fertility workup after HSG (13).

Women with polycystic ovary syndrome (PCOS) who are resistant to clomiphene citrate constitute a patients' group in whom performing laparoscopy and laparoscopic ovarian drilling (LOD) could be a smart treatment option provided that they have good ovarian reserve (Anti-mullerian hormone level >5 ng/ml is agreed by many gynecologists). This option have the advantage of improving the hormonal milieu, inducing mono-follicular growth, avoiding the risk of ovarian hyperstimulation syndrome and minimizing the rate of multiple pregnancy; the risks associated with gonadotropin stimulation (14). Women with mild (minimal) endometriosis may not be diagnosed till diagnostic laparoscopy is performed. Treatment of endometriotic lesions laparoscopically with excision or fulguration is associated with increased pregnancy rates after laparoscopy (15, 16).

The aim of this study was to evaluate the efficacy of laparoscopy to verify causes and management of infertility in our setup.

## Materials and methods

**Figure 1:**
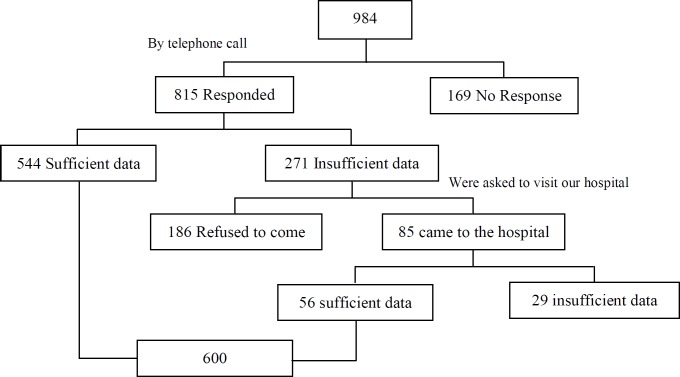
Study flow chart

In this case control study, the medical records of 600 infertile women who were referred to endoscopy unit of Minia University Hospital, Minia, Egypt for laparoscopy or combined laparoscopy and hysteroscopy from January 2012 to December 2014 were reviewed. These women were admitted to the infertility clinic of Minia University Hospital. We tried to approach all women who fulfilled the inclusion criteria for the study through telephone calls. We initially approached 984 women and we got full data from 600 participants who were included in the analysis. The study flowchart is shown in figure 1. 

All data were collected from the Gynecology database which contains data collected from all women undergoing any surgical gynecological procedure in the department. The participants’ files (case notes) were also revised to double check the accuracy of the data registered in the database.

Our inclusion criteria were: infertile women aged between 18 and 40 yr, anovulation due to PCOS, history of tubal factor infertility for example history of previous pelvic operation, history of previous appendectomy, history of puerperal sepsis, or abnormal HSG, history of endometriosis for example dyspareunia, dysmenorrhea or deep pelvic pain, also unexplained infertility. The exclusion criteria were couples with male factor infertility (abnormal semen parameters and/or sexual dysfunctions), women with history of previous laparoscopy, premature ovarian failure, follice stimulating hormone (FSH) <12 IU/L, antral follicle count (AFC) >5, and anti-mullerian hormone (AMH) >0.1 ng/ml.” 

**Follow up**

One yr after laparoscopy, participants were contacted by telephone and asked about the occurrence of pregnancy. Women who got pregnant were asked whether pregnancy was achieved spontaneously, through induction of ovulation, or through ART? They were also asked about the duration interval between laparoscopy and pregnancy, gestational age and pregnancy period. They were also invited to attend the hospital antenatal care clinic for follow up. Participants who didn’t get pregnant were invited to attend the fertility clinic to discuss further steps like the need for ART.

**Ethical consideration**

Ethical approval for the study was obtained from the institutional review board of Obstetrics and Gynecology Department, Faculty of Medicine in Minia University (MUH 7982). Written informed consent was obtained from all participants.

**Statistical analysis**

Data were statistically described in terms of mean±SD (standard deviation) and range for quantitative data, or frequencies and percentages for categorical data. Comparison of age and duration of infertility between primary and secondary infertility cases was done using Student’s *t*-test for independent samples. Agreement between HSG and laparoscopy results was measured using Kappa statistic. p-values less than 0.05 was considered statistically significant. All statistical calculations were done using computer programs SPSS (Statistical Package for the Social Science; SPSS Inc., Chicago, IL, USA) version 21 for Microsoft Windows.

## Results

The study included 600 women who underwent laparoscopy ± hysteroscopy during the study period. The participants' characteristics and pre-operative management are summarized in table I. The causes of infertility as identified by laparoscopy±hysteroscopy were PCOS (25.1%), tubal factor (30%), uterine cause (4%), and endometriosis (2.7%). No cause was identified in 38.2% of cases. Infertility was primary in 344 women and secondary in 256 women. The distribution of the causes of infertility stratified by the type shown in table II. The laparoscopic and hysteroscopic findings in the study population are shown in table III. Different procedures were performed during laparoscopy. LOD for PCO was performed in 152 (25.3%), lysis of intra-peritoneal adhesions in 68 (11.3%), tuboplasty 72 (12%), and ovarian cystectomy in 64 (10.7%), excision of uterine fibroids in 8 (1.3 %), and removal or disconnection of hydrosalpinx in 16 (2.7%) women. Diagnostic laparoscopy was done in 248 cases (41.3%). Operative laparoscopy was performed in 352 cases (58.7%). The total number of women underwent hysteroscopy was 116 cases (19.3%). 

**Figure 2:**
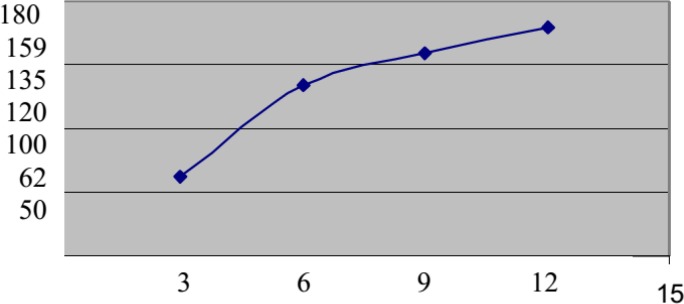
Cumulative pregnancy rate after laparoscopy/hysteroscopy.

The most common procedure performed during hysteroscopy was excision of intrauterine septum. It was done in 24 cases (20.7%) followed by excision of submucous fibroids and excision of intrauterine adhesions; 12 cases each (10.3%). Removal of endometrial polyps was done in four (3.5%) and hysteroscopy in 64 (55.2%) women (Table IV). Based on laparoscopic and hysteroscopic findings, women were treated with different options during the 1 yr after laparoscopy/hysteroscopy. Expectant management was used in 92 women (15.3%). Ovulation induction with anti-estrogens or gonadotropins was used in 372 cases (62%). Twelve cases (2%) underwent further surgical procedure within the 1 yr after laparoscopy (open adhesiolysis (n=5), myomectomy (n=4), and ovarian cystectomy (n=3)). Sixty women (10%) had intrauterine insemination (IUI) and sixty four cases (10.7%) underwent IVF/ICSI treatment. The management options and resultant pregnancy are shown in table V. Within 1 yr after laparoscopy, 180 cases achieved pregnancy (30%). The most favorable outcome was recorded in women with unexplained infertility (36.7% of cases got pregnant) followed by women with PCOS (27.8%). Participants with uterine and tubal factor infertility achieved pregnancy in 25% and 22.8% of cases, respectively. The worst outcome was recorded in women with endometriosis as no pregnancies were achieved in 16 cases diagnosed with laparoscopy to have endometriosis. The number of participants needed to have laparoscopy ± hysteroscopy (Number Needed to Treat (NNT)) to achieve pregnancy within 1 yr was 3.3 (Table VI). The cumulative pregnancy rate after laparoscopy is shown in figure 2.

**Table I:** Participants' characteristics and pre-operative management (n= 600)

**Participants' characteristics**		**Mean** **± SD (range) or n (%)**
Age*		26.1 ± 4.3 (18 – 38)
Parity^#^		
	0	416 (69.4)
	1	108 (18)
	2	68 (11.3)
	≥ 3	8 (1.3)
Type of infertility ^#^		
	Primary	344 (57.3)
	Secondary	256 (42.7)
Duration of infertility (yr)*		4.2 ± 2.5 (2-20)
BMI (kg/m²) *		24.9 ± 2.1 (20.3-31.9)
Previous abdominal/pelvic surgery ^#^		
	No previous surgery	316 (52.7)
	Appendectomy	188 (52.7)
	Cesarean section	88 (14.7)
	Ovarian cystectomy	8 (1.3)
Ultrasound appearance of the ovaries ^#^		
	Definitely PCOS	144 (24)
	Possible PCOS	52 (8.7)
	Definitely non PCOS	404 (67.3)
Hysterosalpingography ^#^		
	Normal	388 (64.7)
	Subnormal	212 (35.3)
	Unilateral tubal obstruction	37 (6.2)
	Bilateral tubal obstruction	93 (15.5)
	Unilateral poor peritoneal spills	15 (2.5)
	Bilateral poor peritoneal spills	33 (5.5)
	Suspected hydroslpinx	20 (3.3)
	Intra-uterine abnormalities	56 (9.3)
Previous treatment ^#^		
	No treatment	132 (22)
	Ovulation induction	468 (78)
	Intrauterine insemination	9 (1.5)
	IVF	3 (0.5)

SD: Standard deviation

BMI: Body mass index

PCOS: Polycystic ovary syndrome

IVF: In vitro fertilization

* Data is presented as mean ± SD (range) and

# n (%).

**Table II:** Frequency of infertility causes according to the infertility type

**Cause**	**Infertility type**		**p-value**
	**Primary** **(n= 344)**	**Secondary** **(n= 256)**	
PCOS (n= 151)	91 (60.3%)	60 (39.7%)	0.01
Tubal factor (n= 180)	96 (53.3%)	84 (46.7%)	0.32
Unexplained (n= 229)	121 (52.8%)	108 (47.2%)	0.17
Uterine factor (n= 24)	20 (83.3%)	4 (16.7%)	0.007
Endometriosis (n= 16)	16 (100%)	0 (0%)	<0.001

Data are presented as n (%).

PCOS: Polycystic ovary syndrome

**Table III:** Laparoscopic and hysteroscopic findings in the study population

**Findings**		**Frequency n (%)**
Right ovary		
	Normal	408 (68)
	Absent (Congenital/surgical)	4 (0.7)
	Atrophic	4 (0.7)
	Polycystic ovary	136 (22.6)
	Ovarian cyst	36 (6)
	Obscured with adhesions	12 (2)
Left ovary		
	Normal	424 (70.6)
	Absent (Congenital/surgical)	0 (0)
	Atrophic	4 (0.7)
	Polycystic ovary	140 (23.3)
	Ovarian cyst	28 (4.7)
	Obscured with adhesions	4 (0.7)
Right fallopian tube		
	Healthy and patent	452 (75.3)
	Absent (Congenital/surgical)	8 (1.3)
	Unhealthy and blocked	60 (10)
	Looks health but blocked	36 (6)
	Obscured with adhesions	24 (4)
	Hydrosalpinx	20 (3.3)
Left fallopian tube		
	Healthy and patent	440 (73.3)
	Absent (Congenital/surgical)	4 (0.7)
	Unhealthy and blocked	80 (13.3)
	Looks health but blocked	36 (6)
	Obscured with adhesions	20 (3.3)
	Hydrosalpinx	20 (3.3)
Uterus by laparoscopy		
	Normal	504 (84)
	Absent or atrophic	8 (1.3)
	Bicornuate	4 (0.7)
	Unicornuate	8 (1.3)
	Arcuate	4 (0.7)
	Broad fundus	20 (3.3)
	Fibroid	24 (4)
	Obscured with adhesions	28 (4.7)
Pouch of Douglas		
	No abnormality detected	536 (89.3)
	Obscured with adhesions	48 (8)
	Spots of endometriosis	16 (2.7)
Abdomen		
	No abnormality detected	536 (89.3)
	Mild / moderate adhesions	36 (6)
	Extensive adhesions	28 (4.7)
Hysteroscopy (n= 116)		
	Normal	64 (55.3)
	Septum	24 (20.7)
	Fibroid	12 (10.3)
	Polyp	4 (3.4)
	Intrauterine adhesions	12 (10.3)

**Table IV:** Procedures are done during laparoscopy/hysteroscopy

**Procedure**		**Frequency n (%)**
Laparoscopy (n= 600)		
	Diagnostic only	248 (41.3)
	Laparoscopic ovarian drilling	152 (25.3)
	Adhesiolysis	68 (11.3)
	Tuboplasty	72 (12)
	Ovarian cystectomy	64 (10.7)
	Excision of fibroids	8 (1.3)
	Removal/disconnection of hydrosalpinx	16 (2.7)
Hysteroscopy (n= 116)		
	Diagnostic only	64 (55.2)
	Excision of septum	24 (20.7)
	Excision of submucous fibroids	12 (10.3)
	Excision of intrauterine adhesions	12 (10.3)
	Removal of polyps	4 (3.5)

**Table V:** Management within 1 yr after laparoscopy/hysteroscopy

**Management**	**Frequency**	**Pregnancy**
Expectant management	92 (15.3)	52.92 (56.5)
Ovulation induction	372 (62)	87.372 (23.4)
Further surgical management	12 (2)	0.12 (0)
Intrauterine insemination (IUI)	60 (10)	3.60 (5)
IVF/ICSI	64 (10.7)	38.64 (59.4)

Data are presented as frequency and percentages

IVF: In vitro fertilization

ICSI: Intracytoplasmic sperm injection

**Table VI:** Pregnancy within 1 yr of laparoscopy/hysteroscopy according to cause of infertility (n= 600)

**Causes of infertility **	**Pregnancy n (%)**	**NNT**
Unexplained (n= 229)	84 (36.7)	2.73
PCOS (n= 151)	42 (27.8)	3.6
Tubal factor (n= 180)	41 (22.8)	4.4
Uterine factor (n= 52)	13 (25)	4s
Endometriosis (n= 16)	0 (0)	

NNT: Number to treat to achieve pregnancy within 1yr after laparoscopy/hysteroscopy

PCOS: Polycystic ovary syndrome

## Discussion

Currently, diagnostic laparoscopy is often bypassed by many infertility specialists in favour of moving forward with ART as the procedure requires general anesthesia and can be associated with a low but potentially serious incidence of risks and complications (17). However, it is believed that diagnostic laparoscopy still has a role in a significant percentage of infertile women (18). In this study, we tried to evaluate the role of laparoscopy/hysteroscopy in diagnosis and management of infertility in our setup. In the current study, tubal pathology was found in 30% of cases. This high incidence reflects the high rate of pelvic infection in our setting. These results are similar to those quoted from other studies (19-22). A higher rate is quoted from one study; 62.8% in women with primary infertility and 54.8% in women with secondary infertility (18). While, lower rates were found by Bonneau and colleagues (18.9%) and Siam (14.4%) (23, 24).

PCOS represented 25.1% of cases which is similar to the rates quoted from other studies done on infertile women in Egypt (24, 25). The much lower rate was reported by Geetika and co-workers (18). This can be attributed to the genetic theory of the pathogenesis of PCOS which can explain the different incidence according to the geographical distribution (26). Among 600 cases, the septate or subseptate uterus was found in 24 cases, bicornuate uterus in 8 cases, unicornuate uterus in 4 cases and absent or atrophic uterus in 8 cases. This rate reflects the high prevalence of müllerian duct anomalies in our locality. This is in agreement with what was reported by El Saman *et al* (27). The prevalence of unexplained infertility in this study was 38% which is quite high. This can be explained by including of women with normal investigations for infertility after 12 months only. It is supposed that if these women are treated expectantly for a longer time, it is possible that the prevalence of unexplained infertility would be lower. Endometriosis was identified in 2.7% of cases; all of them were primary infertility. This rate is much lower than the rates reported by Bonneau *et al* (75.8%) and Meuleman *et al* (78%) (23, 28). This difference can be explained also on the basis of the genetic origin of disease (29). 

Participants received different types of treatment within 1 yr of laparoscopy. Expectant management was done in 15.3% of cases and achieved pregnancy in 56.5% of them. Most of these women had no cause for infertility identified by laparoscopy/ hysteroscopy. This high pregnancy rate may reflect that in women with unexplained infertility, it may be better to treat them expectantly for a longer time before deciding to perform a laparoscopy. 

Ovulation induction with anti-estrogens or gonadotropins was received in 62% of cases and 23.4% achieved pregnancy on top of ovulation induction. Most of these women were PCOS and that reflects improvement of ovarian responsiveness to ovulation induction after LOD. IUI was tried in 10% of cases but low pregnancy rate was achieved (5%). IVF/ICSI was done in 10.7% of cases and the pregnancy rate was 59.4%. This high rate may be due to the identification of women with tubal pathology who were in need of IVF/ICSI treatment and tubal removal or disconnection in cases of hydrosalpinx who underwent IVF after laparoscopy.

## Conclusion

In conclusion, laparoscopy still has an important role in the diagnosis and treatment of infertility. A significant number of infertile women, such as those with a tubal factor, PCOS, and women with unexplained infertility can benefit from it. Operative procedures at the time of laparoscopy can enhance conception, naturally, or with IUI/IVF, such as lysis of adhesions, ablation of endometriosis, and salpingectomy or disconnection for hydrosalpinx.
